# Supplementary material for: Enhancement of electrocatalytic oxygen evolution by chiral molecular functionalization of hybrid 2D electrodes
Source: Nat Commun. 2022 Jun 10;13:3356. doi: 10.1038/s41467-022-31096-8 (PMC9187664; doi:10.1038/s41467-022-31096-8)
Supplement: Supplementary file 1 — Supplementary Information [file 41467_2022_31096_MOESM1_ESM.pdf]

Supplementary information

Enhancement of Electrocatalytic Oxygen Evolution by Chiral Molecular Functionalization of Hybrid 2D Electrodes

Yunchang Liang,[1,2,*] Karla Banjac,[1,2] Kévin Martin,[3] Nicolas Zigon,[3] Seunghwa Lee,[4] Nicolas Vanthuyne,[5] Felipe Andres Garcés-Pineda,[6] José R. Galán-Mascarós,[6,7] Xile Hu,[4] Narcis Avarvari,[3,*] Magalí Lingenfelder[1,2,*]

[1] Max Planck-EPFL Laboratory for Molecular Nanoscience and Technology, École Polytechnique Fédérale de Lausanne (EPFL), 1015 Lausanne, Switzerland

[2] Institut of Physics (IPHYS), Ecole Polytechnique Fédérale de Lausanne (EPFL), 1015 Lausanne, Switzerland

[3] Univ Angers, CNRS, MOLTECH-Anjou, SFR MATRIX, F-49000 Angers, France

[4] Laboratory of Inorganic Synthesis and Catalysis, Institute of Chemical Sciences and Engineering, École Polytechnique Fédérale de Lausanne (EPFL), 1015 Lausanne, Switzerland

[5] Aix Marseille Université, CNRS, Centrale Marseille, iSm2, Marseille, France

[6] Institute of Chemical Research of Catalonia (ICIQ), The Barcelona Institute of Science and Technology (BIST), Av. Països Catalans 16, E-43007 Tarragona, Spain

[7] Catalan Institution for Research and Advanced Studies (ICREA), Passeig Lluis Companys, 23, Barcelona 08010, Spain

*e-mail: yunchang.liang@epfl.ch; narcis.avarvari@univ-angers.fr; magali.lingenfelder@epfl.ch

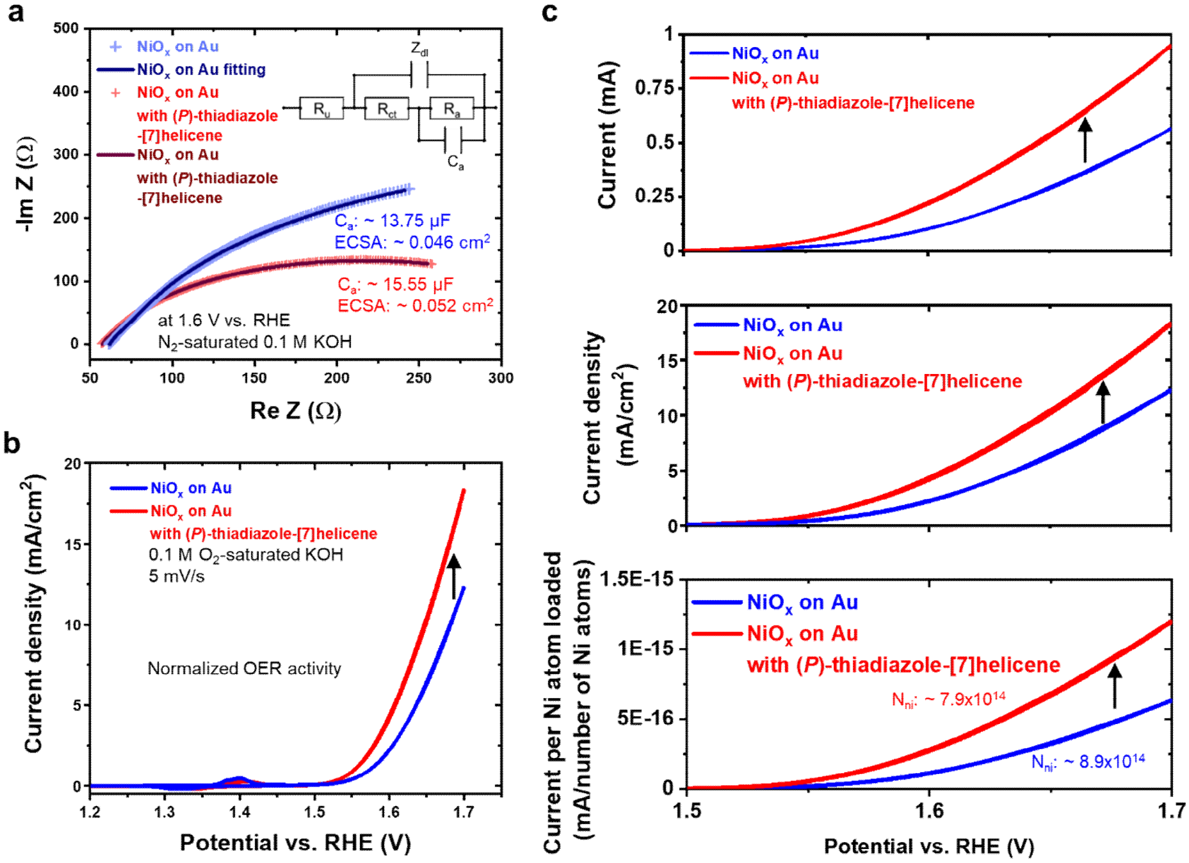

Supplementary Figure 1 | Chiral Molecular Functionalization effect on the current density. a, impedance spectroscopy of the NiO_x on Au surface before and after the deposition of (*P*)-thiadiazole-[7]helicene and the data-fitting results. Inset is the equivalent electric circuit where R_u is the uncompensated resistance, Z_{dl} is the double layer impedance, R_{ct} is the charge transfer resistance, R_a is the adsorption resistance and C_a is the adsorption capacitance. **b**, the activity of the NiO_x sample normalized by the ECSA obtained from EIS measurements. **c**, comparison of the overall current, the specific current density obtained using the ECSA determined by the EIS method and the current per Ni atom loaded. The number of Ni atoms was calculated by integrating the area between the Ni oxidation peak and a line fit described by the Butler–Volmer equation at low overpotentials described elsewhere[1], assuming the oxidation is a single electron transfer reaction. Clear activity enhancement can be seen in all three cases. The slight change in the number of Ni atoms is likely due to the detachment of NiO_x islands during the removal of excessive helicene molecules and catalyst losses during the long-term OER measurements, as reported by Farhat et al.[2] and Chung et al.[3]. Source data are provided as a Source Data file.

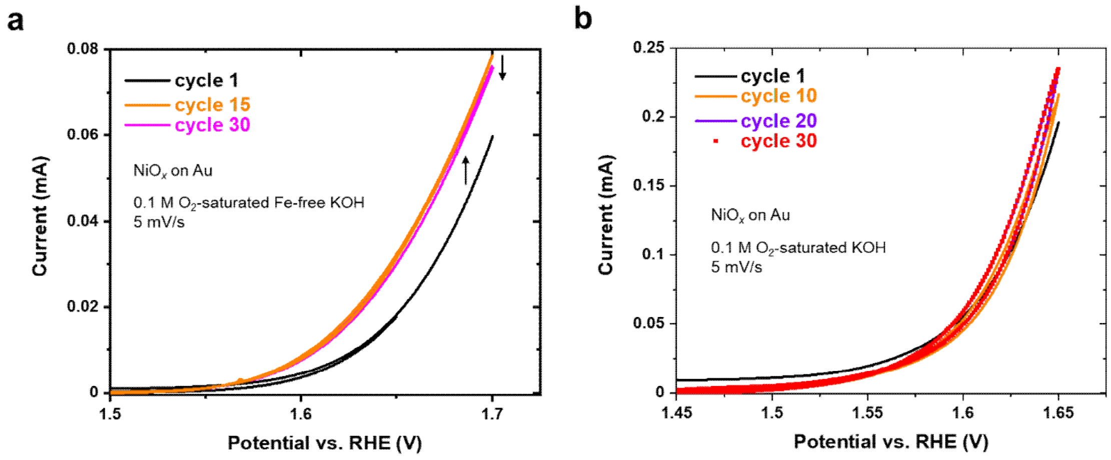

Supplementary Figure 2 | Activity change of freshly prepared electrodes. Activity change of freshly prepared NiO_x on Au in **a** Fe-free and **b** unpurified KOH. Source data are provided as a Source Data file.

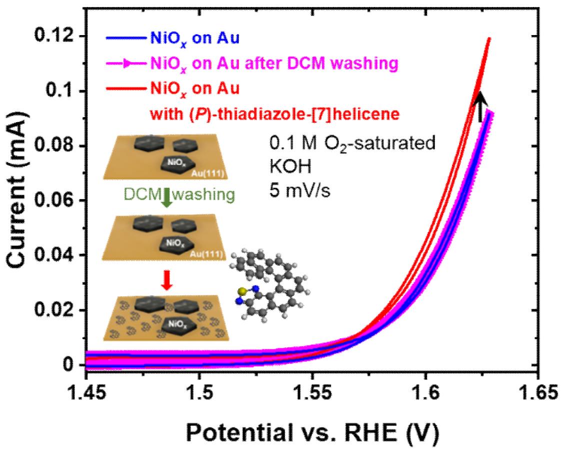

Supplementary Figure 3 | Effect of DCM washing. Activity of bare NiO_x on Au, after pure DCM washing and after (*P*)-thiadiazole-[7]helicene deposition in 0.1 M O_2-saturated KOH. A Ag/AgCl (3 M NaCl) RE was used. The potential has been converted to the RHE scale. Source data are provided as a Source Data file.

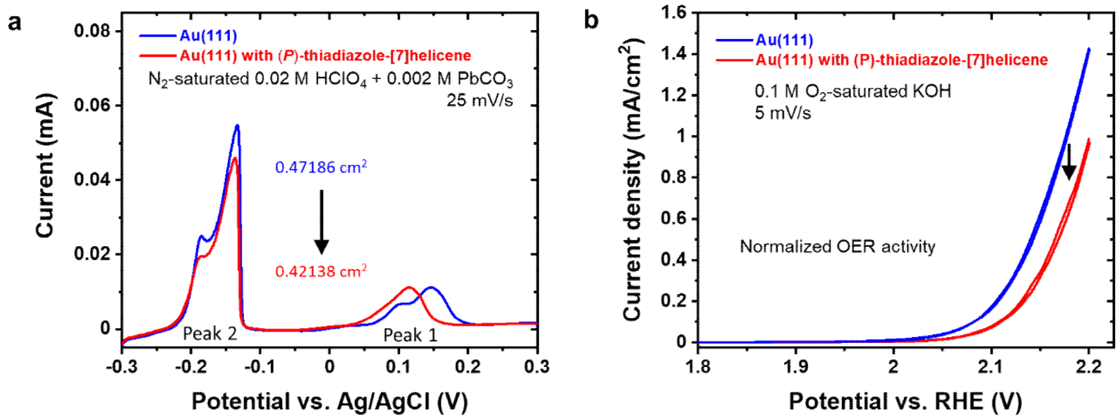

Supplementary Figure 4 | Chiral molecule deposition effect on the ECSA of Au. a, Pb stripping on Au surfaces before and after (*P*)-thiadiazole-[7]helicene deposition and **b**, normalized OER activity[4,5]. Source data are provided as a Source Data file.

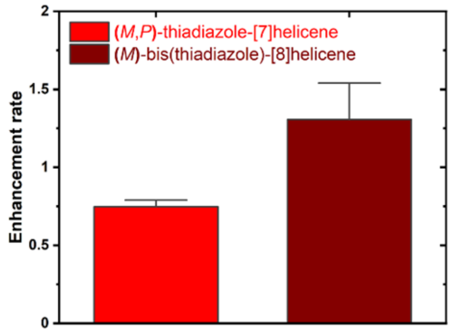

Supplementary Figure 5 | Activity enhancement comparison between different chiral molecules. OER activity enhancement rate (i.e., $(i_{with\ helicene} - i_{without\ helicene})/i_{without\ helicene}$) at 1.65 V vs. RHE by thiadiazole-[7]helicene and (*M*)-bis(thiadiazole)-[8]helicene from five nominally identical NiO_x electrodes, respectively. The error bars show the standard deviation. The mean enhancement rates are 75 ± 26 % and 131 ± 23 %, respectively (descriptive statistics). The deviation is likely caused by the different catalyst loadings, mass transfer processes in long-term measurements (e.g., O_2 gas bubble formation) and the different amounts of chiral molecules that can effectively affect the OER activity of the NiO_x islands on different samples. Source data are provided as a Source Data file.

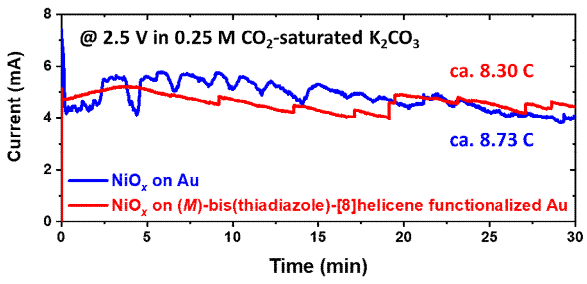

Supplementary Figure 6 | Chronoamperometry measurements for selectivity comparison. Chronoamperometry (30 min) of NiO_x on Au and NiO_x on monolayer (*M*)-bis(thiadiazole)-[8]helicene functionalized Au at 2.5 V vs. RHE in 0.25 M CO_2 saturated K_2CO_3. The total charges transferred were approximately 8.73 C and ca. 8.30 C, respectively. Source data are provided as a Source Data file.

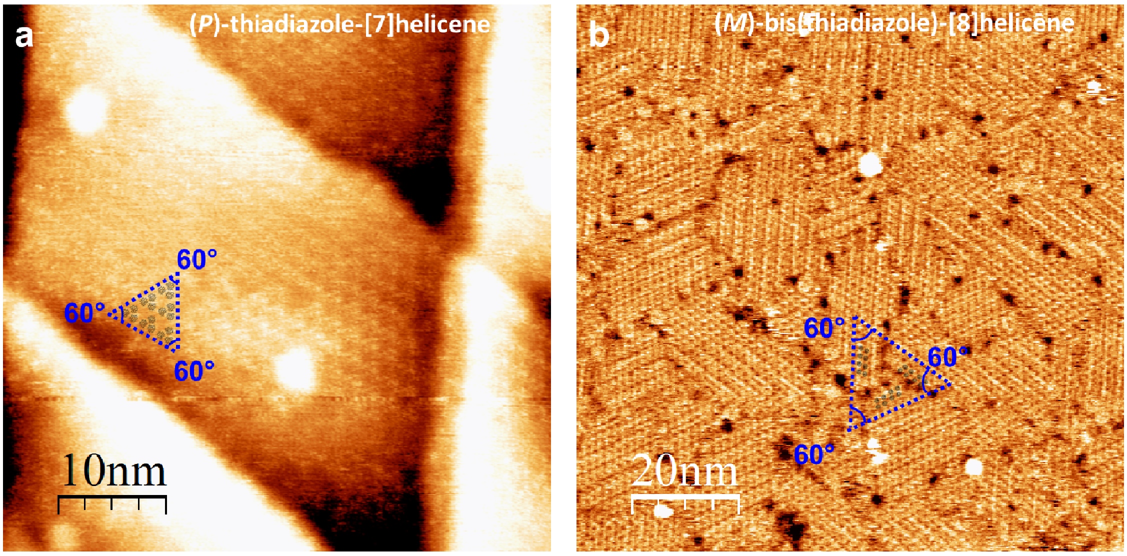

Supplementary Figure 7 | STM images of SAMs. STM images of the SAM of **a** (*P*)-thiadiazole-[7]helicene as packed trimers (image size: 50 nm x 50 nm, tunneling current: 15 pA, sample bias: 50 mV) and **b** (*M*)-bis(thiadiazole)-[8]helicene with three rotational domains 60° to each other (image size: 100 nm x 100 nm, tunneling current: 20 pA, sample bias: 50 mV).

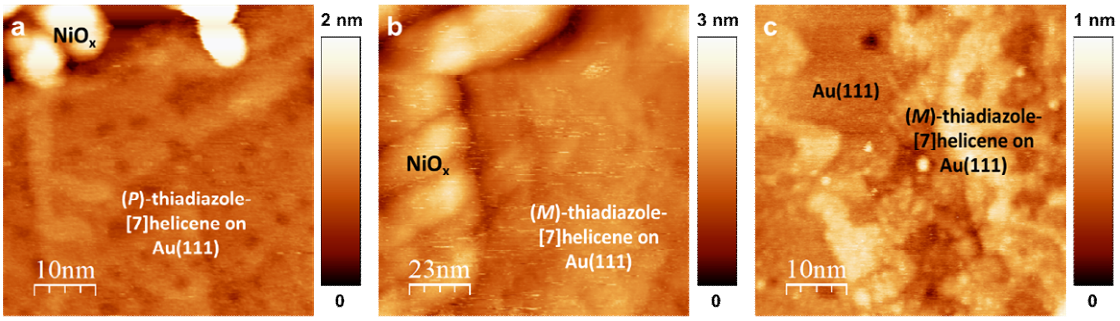

Supplementary Figure 8 | STM images of electrodes after the OER. STM images (image size: 50 nm x 50 nm) of **a** (*P*)- and **b**,**c** (*M*)-thiadiazole-[7]helicene functionalized electrode surface after the OER measurements. All images were recorded in air. Imaging parameters: **a**, tunneling current: 40 pA, sample bias: -500 mV; **b**, tunneling current: 35 pA, sample bias: -316.8 mV; **c**, tunneling current: 30 pA, sample bias: -316.8 mV.

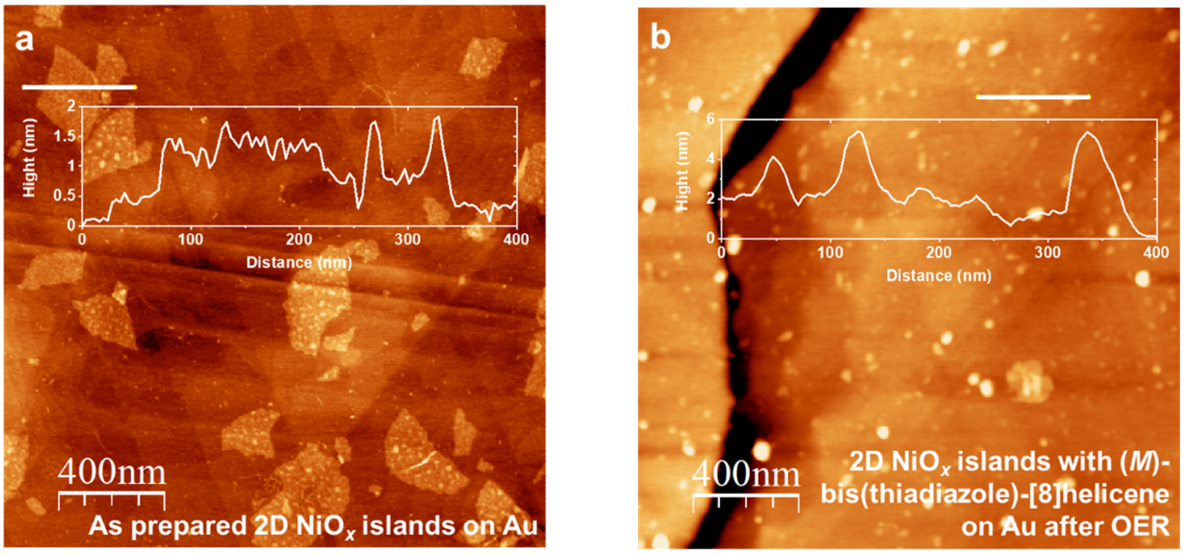

Supplementary Figure 9 | AFM images of the NiO$_x$ islands on Au. AFM images of **a** freshly prepared NiO$_x$ islands on Au and **b** NiO$_x$ islands with (*M*)-bis(thiadiazole)-[8]helicene on Au after the OER. Source data are provided as a Source Data file.

Experimental Section

All the solvents and precursors for the synthesis of the helicenes were commercially available and used without further purification. ^1H and ^{13}C spectra were recorded on a Bruker Advance DRX 300 spectrometer operating at 300 MHz for ^1H and 76 MHz for ^{13}C. Chemical shifts are given in ppm relative to tetramethylsilane TMS and coupling constants *J* in Hz. The residual non-deuterated solvent was used as an internal standard. Mass spectra were obtained by the MALDI-TOF techniques by using a Bruker Biflex-IIITM apparatus, equipped with a 337 nm N_2 laser. 2,7-bis((triphenylphosphonium)methyl)naphthalene bromide **1** and [7]helicene-thiadiazole were synthesized according to published literature protocols[6,7]. 2,1,3-benzothiadiazole-5-carbaldehyde was purchased from ABCR chemicals, and *n*-BuLi and I_2 from Sigma Aldrich, and used as received.

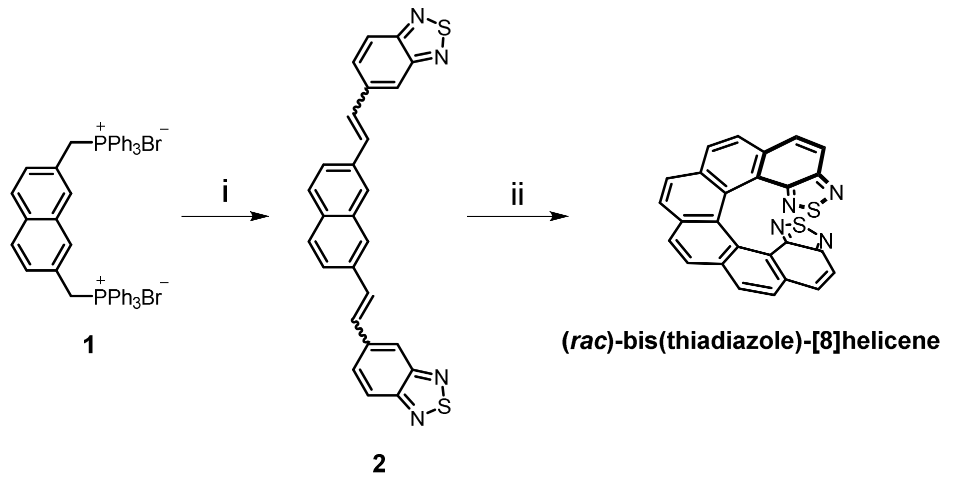

Supplementary Figure 10 | Synthesis of (*rac*)-bis(thiadiazole)-[8]helicene. i) benzothiadiazole-5-carbaldehyde, *n*-BuLi, THF, -78 °C to RT; ii) I_2 (cat.), hv, toluene, RT.

Crystallography

X-Ray structure determinations, details about data collection and solution refinement are given in Table S2. Data collections were performed on a Rigaku Oxford Diffraction SuperNova diffractometer equipped with an Atlas CCD detector and micro-focus Cu-K_α radiation (λ = 1.54184 Å). The structures were solved by intrinsic phasing and refined on F^2 by full matrix least-squares techniques with SHELX programs (SHELXT 2018/2 and SHELXL 2018/3)[8,9] using the ShelXle and the Olex2 graphical user interfaces[10,11]. All non-H atoms were refined anisotropically, and absorption was corrected by multiscan empirical absorption using spherical harmonics with the CrysAlisPro program. The H atoms were placed at calculated positions and refined using a riding model. Crystallographic data for the three structures have been deposited with the Cambridge Crystallographic Data Centre, deposition numbers CCDC 2109360 for (*rac*)-, 2109361 for (*P*)- and 2109362 for (*M*)-bis(thiadiazole)-[8]helicene. These data can be obtained free of charge from CCDC, 12 Union road, Cambridge CB2 1EZ, UK (e-mail: deposit@ccdc.cam.ac.uk or http://www.ccdc.cam.ac.uk).

Supplementary Table 1 | Crystallographic data, details of data collection and structure refinement parameters for the compounds (*rac*)-, (*P*)- and (*M*)-bis(thiadiazole)-[8]helicene.

	**(*rac*)-bis(thiadiazole)-[8]helicene**	**(*P*)-bis(thiadiazole)-[8]helicene**	**(*M*)-bis(thiadiazole)-[8]helicene**
Formula sum	$C_{26}H_{12}N_4S_2$	$C_{26}H_{12}N_4S_2$	$C_{26}H_{12}N_4S_2$
Formula weight	444.52	444.52	444.52
Crystal system	triclinic	monoclinic	monoclinic
Space group	*P*-1	$P2_1$	$P2_1$
a/Å	8.0568(3)	9.5161(2)	9.5191(2)
b/Å	11.0813(5)	11.7193(2)	11.7168(2)
c/Å	11.5581(5)	9.7503(2)	9.7410(2)
α/°	81.947(4)	90	90
β/°	78.412(3)	114.570(3)	114.668(2)
γ/°	72.829(4)	90	90
V/Å^3	962.21(7)	988.92(4)	987.30(4)
Z	2	2	2
D_c/g cm^{-3}	1.534	1.493	1.495
T/K	150.00(10)	150.00(10)	150.00(10)
μ/mm^{-1}	2.697	2.625	2.629
Reflections collected	6629	19261	17010
Independent reflection	3739	4073	4024
final R_1^a, wR_2^b [$I > 2\sigma(I)$]	0.0548/0.1530	0.0293, 0.0792	0.0261 , 0.0672
R_1^a,wR_2^b (all data)	0.0554/0.1541	0.0297, 0.0797	0.0266, 0.0678
goodness-of-fit on F^2	1.034	1.034	1.064
$\Delta\rho_{min}/\Delta\rho_{max}$ (e Å^{-3})	-0.355/0.550	-0.275/0.172	-0.232/0.168
Completeness (%)	99.54	99.89	99.79
Flack parameter	/	0.017(8)	-0.006(8)
CCDC number	2109360	2109361	2109362

$^aR_1 = \sum||F_o|-|F_c||/\sum|F_o|$. $^bwR_2 = [\sum w(F_o^2 - F_c^2)^2/\sum w(F_o^2)^2]^{1/2}$; $w = 1/[\sigma^2(F_0^2)+(aP)^2+bP]$ where $P= [max(F_0^2, 0) + 2F_c^2]/3$.

Supplementary Table 2 | Selected bond distances (Å) and dihedral angle between two terminal helicene planes for compounds (*rac*)-, (*P*)- and (*M*)-bis(thiadiazole)-[8]helicene.

**(*rac*)-bis(thiadiazole)-[8]helicene**			**(*P*)-bis(thiadiazole)-[8]helicene**			**(*M*)-bis(thiadiazole)-[8]helicene**		
S1	N2	1.6175(16)	S001	N003	1.6140(19)	S001	N003	1.613(2)
S1	N1	1.6200(18)	S001	N004	1.617(2)	S001	N004	1.619(1)
S2	N3	1.6137(15)	S002	N005	1.614(2)	S002	N005	1.617(2)
S2	N4	1.621(2)	S002	N007	1.624(3)	S002	N006	1.623(3)
N2	C2	1.340(2)	N003	C006	1.340(3)	N003	C007	1.341(2)
N3	C23	1.341(2)	N004	C008	1.340(3)	N004	C008	1.339(3)
N1	C1	1.342(3)	N005	C009	1.337(3)	N005	C009	1.338(3)
N4	C24	1.333(3)	C006	C008	1.435(3)	N006	C00M	1.337(3)
C2	C1	1.439(3)	C006	C00E	1.452(3)	C007	C008	1.435(3)
C2	C3	1.445(3)	N007	C00O	1.336(4)	C007	C00D	1.447(3)
C23	C19	1.441(3)	C008	C00K	1.429(3)	C008	C00I	1.432(3)
C23	C24	1.434(3)	C009	C00B	1.452(3)	C009	C00C	1.449(2)
C1	C6	1.426(3)	C009	C00O	1.442(3)	C009	C00M	1.441(4)
C3	C7	1.445(3)	C00A	C00C	1.449(3)	C00A	C00B	1.451(2)
C3	C4	1.405(3)	C00A	C00D	1.447(3)	C00A	C00D	1.439(3)
C15	C19	1.438(3)	C00A	C00J	1.417(3)	C00A	C00L	1.417(4)
C15	C11	1.442(3)	C00B	C00C	1.439(3)	C00B	C00E	1.447(3)
C15	C16	1.422(3)	C00B	C00N	1.410(3)	C00B	C00G	1.414(3)
C7	C11	1.447(3)	C00C	C00I	1.423(3)	C00C	C00E	1.440(3)
C7	C8	1.419(3)	C00D	C00E	1.437(3)	C00C	C00N	1.410(4)
C19	C20	1.416(3)	C00D	C00F	1.422(3)	C00D	C00K	1.411(2)
C24	C25	1.429(3)	C00E	C00G	1.412(3)	C00E	C00F	1.422(2)
C4	C5	1.439(3)	C00F	C00S	1.426(4)	C00F	C00J	1.426(4)
C4	C10	1.428(3)	C00F	C00T	1.408(4)	C00F	C00P	1.419(3)
C11	C12	1.419(3)	C00G	C00R	1.425(3)	C00G	C00H	1.430(3)
C6	C5	1.352(3)	C00G	C00W	1.436(3)	C00G	C00O	1.423(4)

C16	C22	1.416(4)	C00H	C00I	1.427(4)	C00H	C00J	1.343(4)
C16	C17	1.424(4)	C00H	C00L	1.346(4)	C00I	C00V	1.349(4)
C20	C26	1.445(4)	C00I	C00M	1.418(4)	C00K	C00U	1.424(3)
C20	C21	1.421(3)	C00J	C00L	1.429(4)	C00K	C00V	1.437(3)
C8	C9	1.410(4)	C00J	C00Q	1.420(4)	C00L	C00S	1.412(3)
C8	C14	1.443(4)	C00K	C00W	1.347(4)	C00L	C00T	1.427(4)
C9	C10	1.364(4)	C00M	C00P	1.355(4)	C00M	C00R	1.426(5)
C14	C13	1.348(4)	C00N	C00P	1.417(4)	C00N	C00Q	1.417(3)
C22	C21	1.336(4)	C00N	C00V	1.443(4)	C00N	C00W	1.445(4)
C12	C18	1.425(4)	C00O	C00U	1.427(4)	C00O	C00T	1.352(3)
C12	C13	1.414(4)	C00Q	C00S	1.352(4)	C00P	C00Q	1.357(4)
C25	C26	1.332(4)	C00R	C00T	1.355(4)	C00R	C00W	1.348(3)
C17	C18	1.348(4)	C00U	C00V	1.342(5)	C00S	C00U	1.356(4)
Dihedral angle between the planes C1-C2-N2-S1-N1 and C24-C23-N3-S2-N4		29.37	Dihedral angle between the planes N003-C006-C008-N004-S001 and N005-S002-N007-C00O-C009		35.02	Dihedral angle between the planes C007-C008-N004-S001-N003 and C009-N005-S002-N006-C00M		35.15

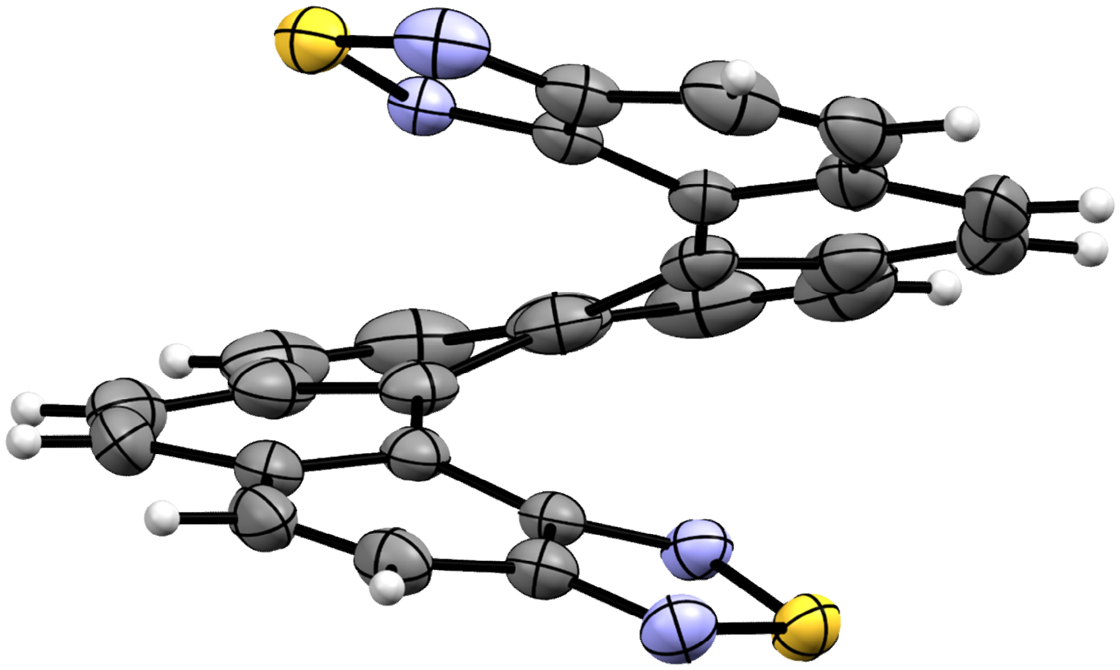

Supplementary Figure 11 | (*rac*)-bis(thiadiazole)-[8]helicene. Asymmetric unit of the X-Ray structure of (*rac*)-bis(thiadiazole)-[8]helicene.

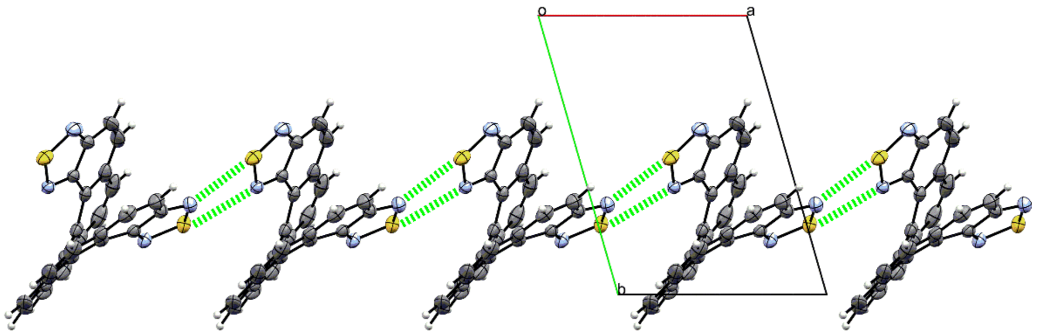

Supplementary Figure 12 | (*rac*)-bis(thiadiazole)-[8]helicene. Homochiral *M* columnar stacks in the structure of (*rac*)-bis(thiadiazole)-[8]helicene viewed along the *c* axis. N-S short contacts (3.014(2) Å and 3.103(2) Å) are highlighted in green.

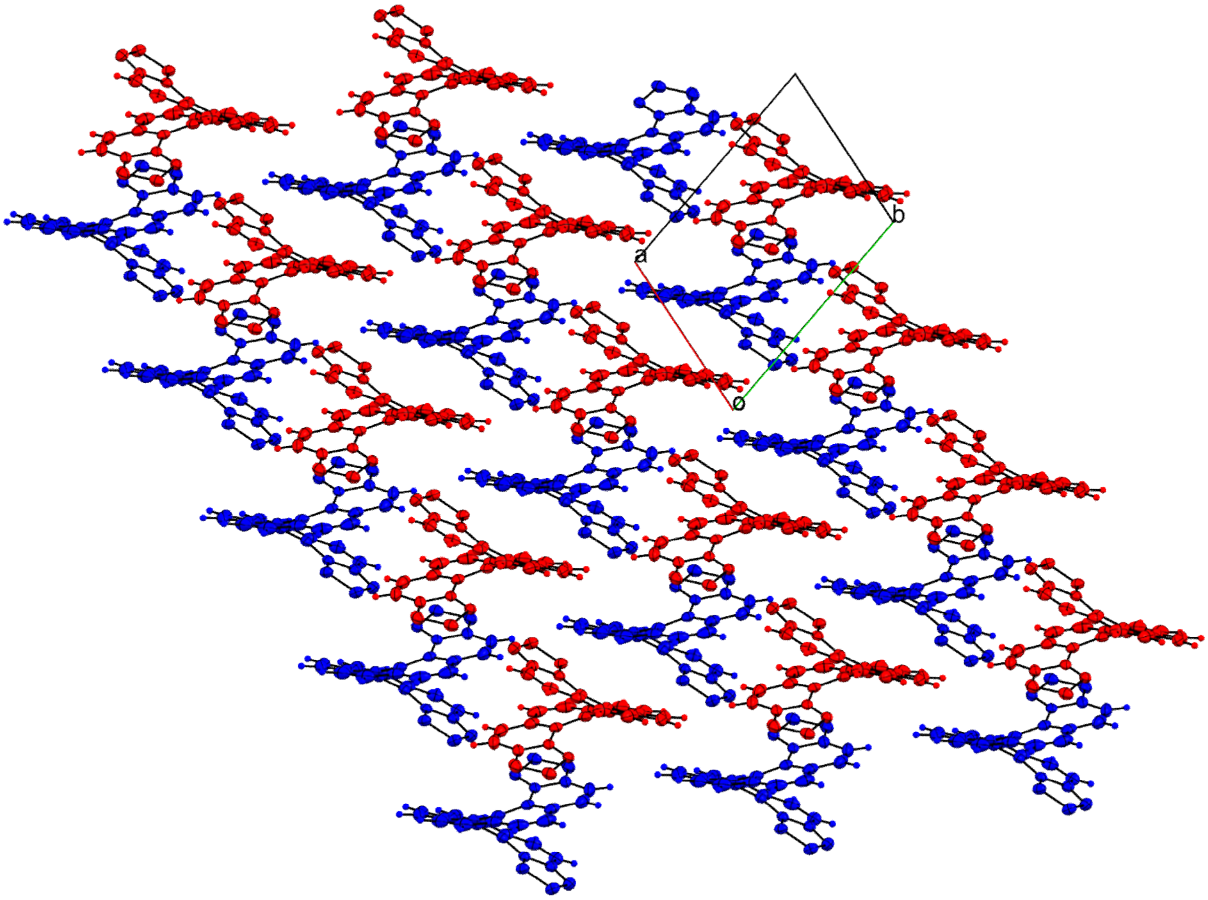

Supplementary Figure 13 | (*rac*)-bis(thiadiazole)-[8]helicene. Packing of (*rac*)-bis(thiadiazole)-[8]helicene viewed along the *c* axis (blue: *P* enantiomer; red: *M* enantiomer).

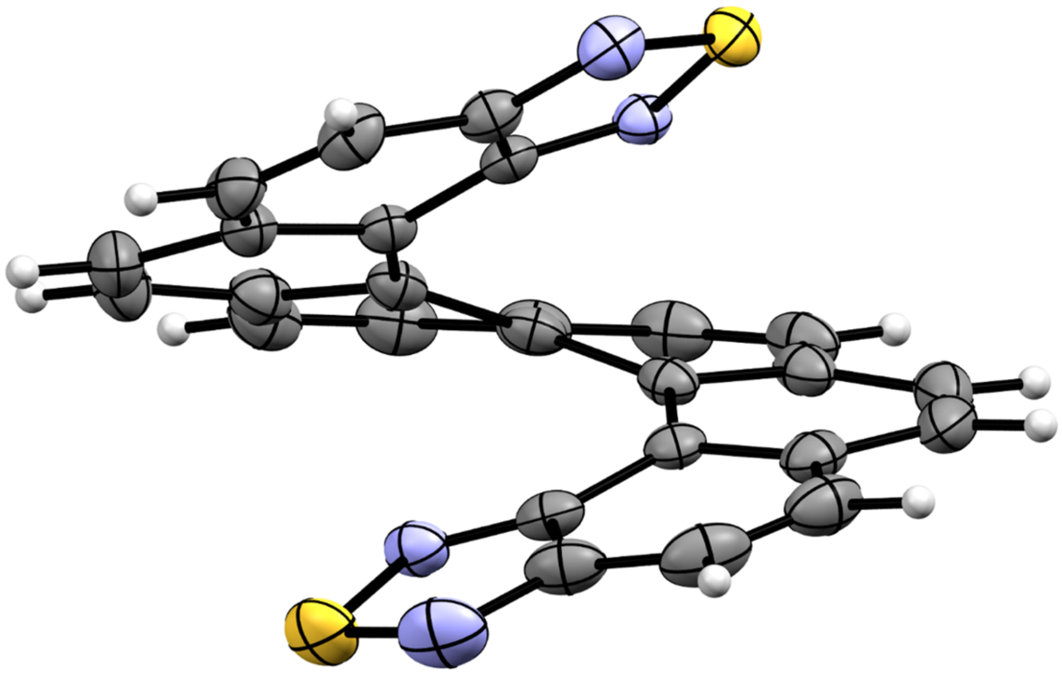

Supplementary Figure 14 | (*P*)-bis(thiadiazole)-[8]helicene. Asymmetric unit of the X-Ray structure of (*P*)-bis(thiadiazole)-[8]helicene.

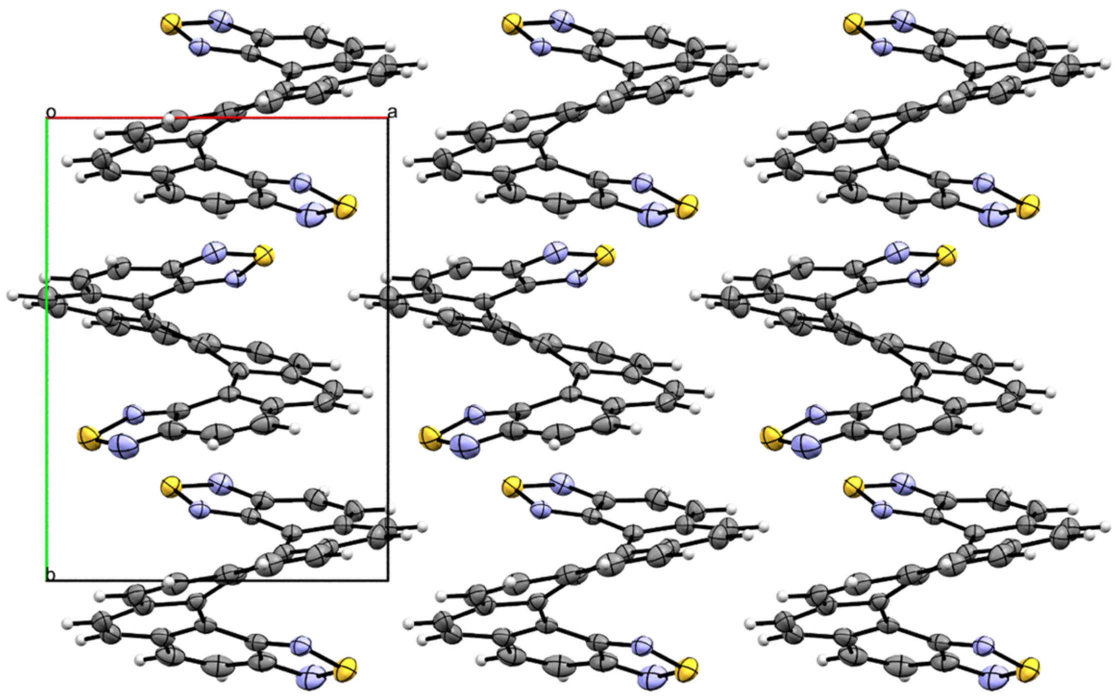

Supplementary Figure 15 | (*P*)-bis(thiadiazole)-[8]helicene. Packing of (*P*)-bis(thiadiazole)-[8]helicene viewed along the *c* axis.

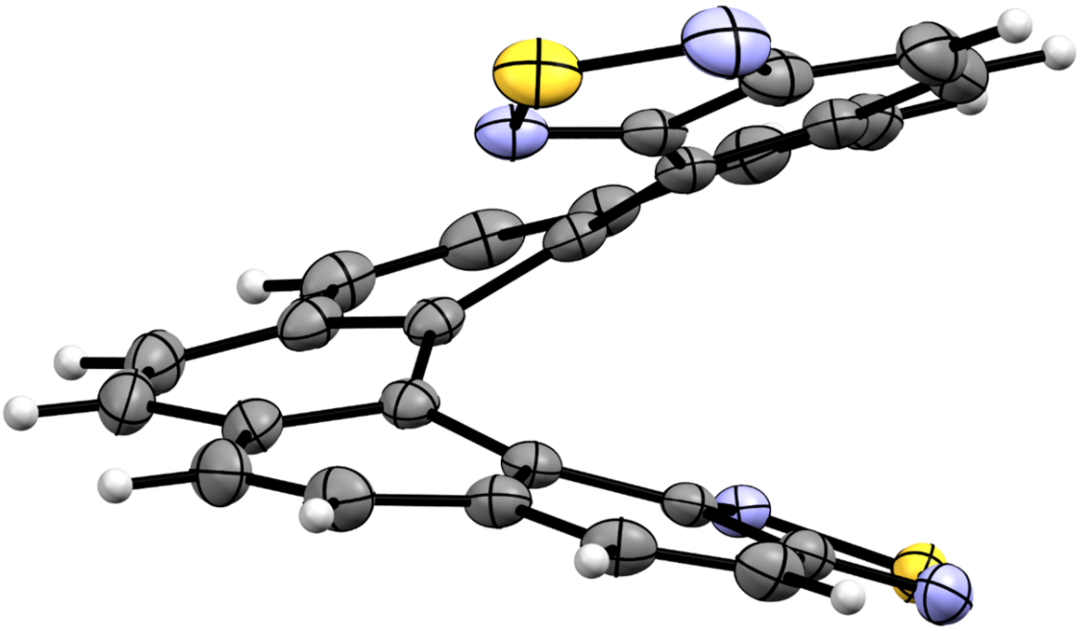

Supplementary Figure 16 | (*M*)-bis(thiadiazole)-[8]helicene. Asymmetric unit of the X-Ray structure of (*M*)-bis(thiadiazole)-[8]helicene.

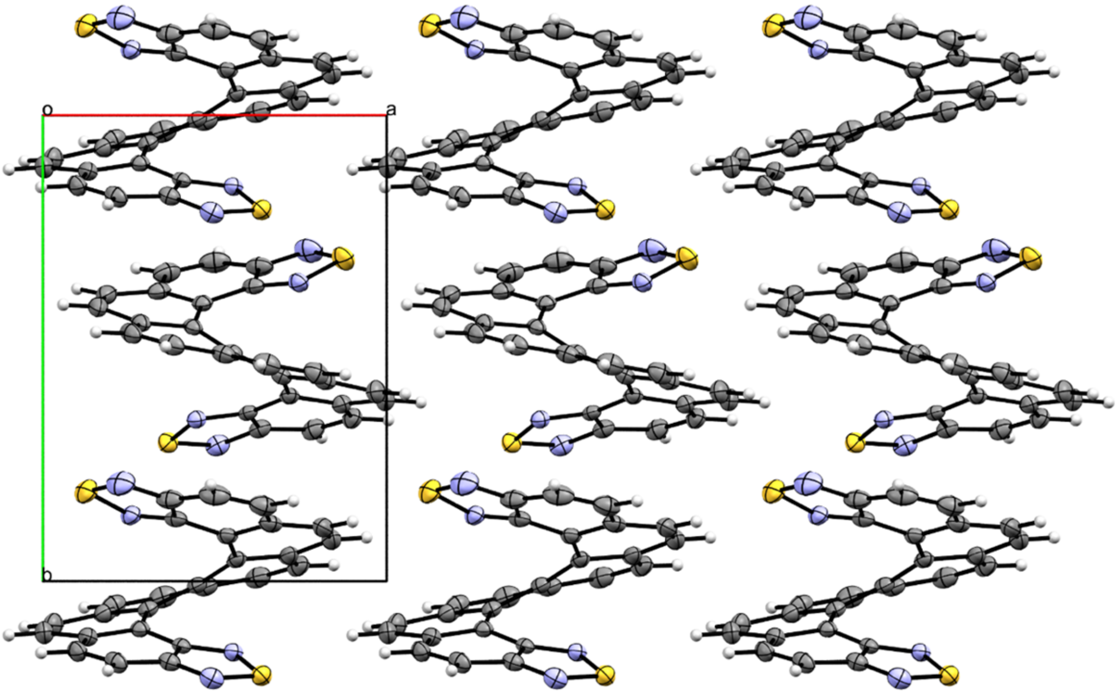

Supplementary Figure 17 | (*M*)-bis(thiadiazole)-[8]helicene. Packing of (*M*)-bis(thiadiazole)-[8]helicene viewed along the *c* axis.

Chiral resolution on HPLC

Analytical chiral HPLC separation for (*rac*)-(bis)thiadiazole-[8]helicene

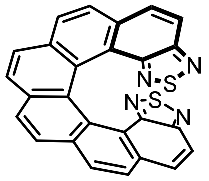

• The sample was dissolved in a mixture of heptane, dichloromethane and 2-PrOH, injected on the chiral column, and detected with a UV detector at 230 nm and a circular dichroism detector at 254 nm. The flow-rate was 1 mL/min.

Column	Mobile Phase	t_1	k_1	t_2	k_2	α	Rs
**Chiralpak IF**	Heptane / 2-PrOH / dichloromethane (80/10/10)	5.58 (+)	0.89	6.72 (-)	1.28	1.43	4.54

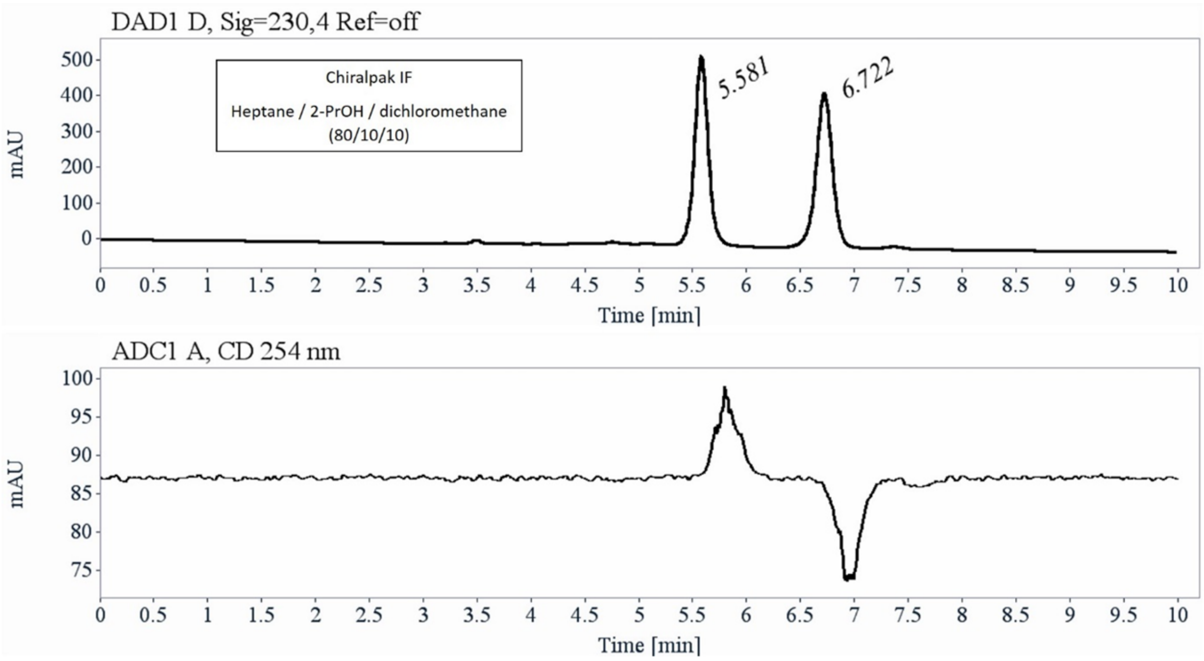

RT [min]	Area	Area%	Capacity Factor	Enantioselectivity	Resolution (USP)
5.58	4758	50.61	0.89		
6.72	4643	49.39	1.28	1.43	4.54
Sum	9402	100.00			

Preparative separation for (*rac*)-(bis)thiadiazole-[8]helicene

• Sample preparation: About 28 mg of compound are dissolved in 4 mL of dichloromethane.

• Chromatographic conditions: Chiralpak IF (250 x 10 mm), hexane / 2-PrOH / dichloromethane (80/10/10) as mobile phase, flow-rate = 5 mL/min, UV detection at 230 nm.

• Injections (stacked): 27 times 150 µL, every 8 minutes.

• First fraction: 7 mg of the first eluted with *ee* > 99.5%

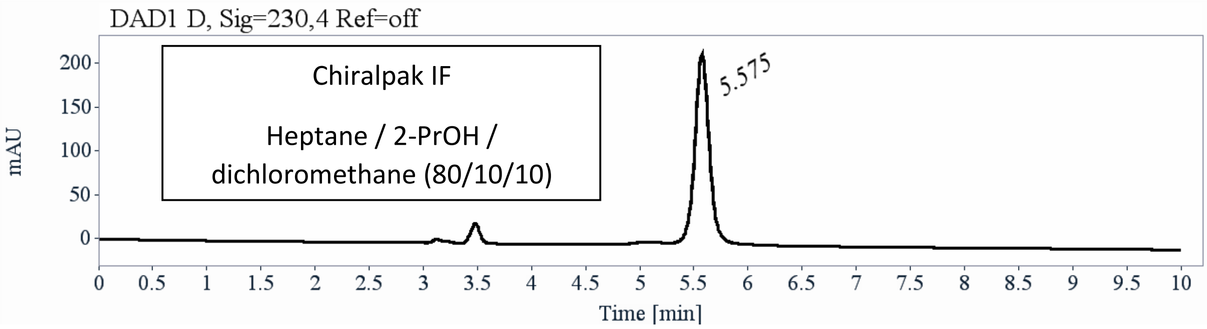

• Second fraction: 6.9 mg of the second eluted with *ee* > 99.5 %

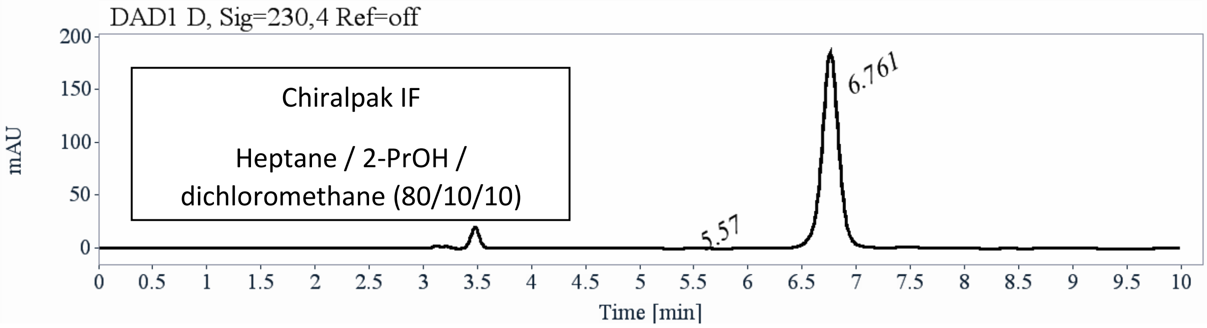

Optical rotations

Optical rotations were measured on a Jasco P-2000 polarimeter with a halogen lamp (589 nm, 578 nm and 546 nm), in a 10 cm cell, thermostated at 25 °C with a Peltier controlled cell holder.

λ (nm)	*P* enantiomer, first eluted on Chiralpak IF<br>$[\alpha]_\lambda^{25}$ (CH_2Cl_2, c =0.01)	*M* enantiomer, second eluted on Chiralpak IF<br>$[\alpha]_\lambda^{25}$ (CH_2Cl_2, c =0.01)
589	+ 8100	- 8100
578	+ 8900	- 8900
546	+ 12600	- 12600

Electronic Circular Dichroism

ECD and UV spectra were measured on a JASCO J-815 spectrometer equipped with a JASCO Peltier cell holder PTC-423 to maintain the temperature at 25.0 ± 0.2 °C. A CD quartz cell of 1 mm of optical pathlength was used. The CD spectrometer was purged with nitrogen before recording each spectrum, which was baseline subtracted.

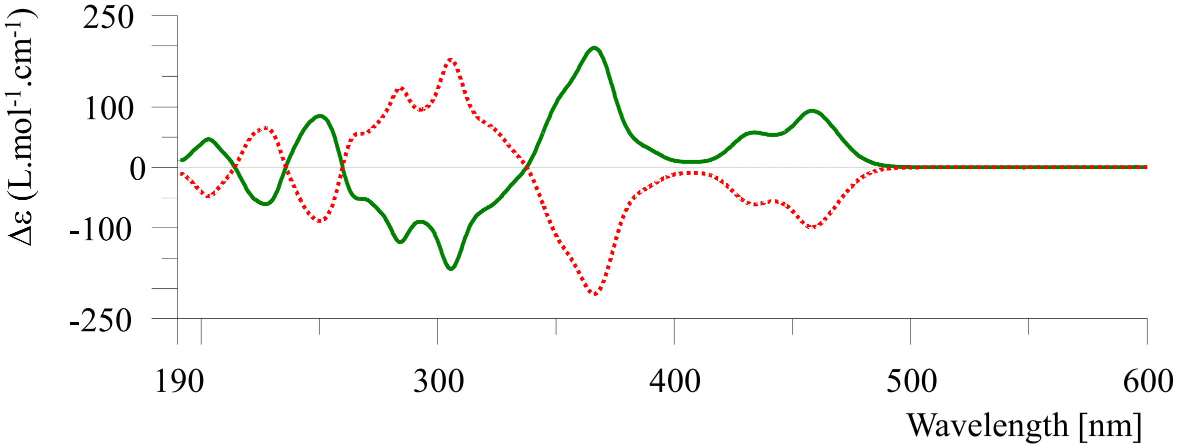

Supplementary Figure 18 | CD spectroscopy. ECD spectra for *P* enantiomer (green solid line) and *M* enantiomer (red dotted line) in CH_3CN (0.216 mM, 298 K).

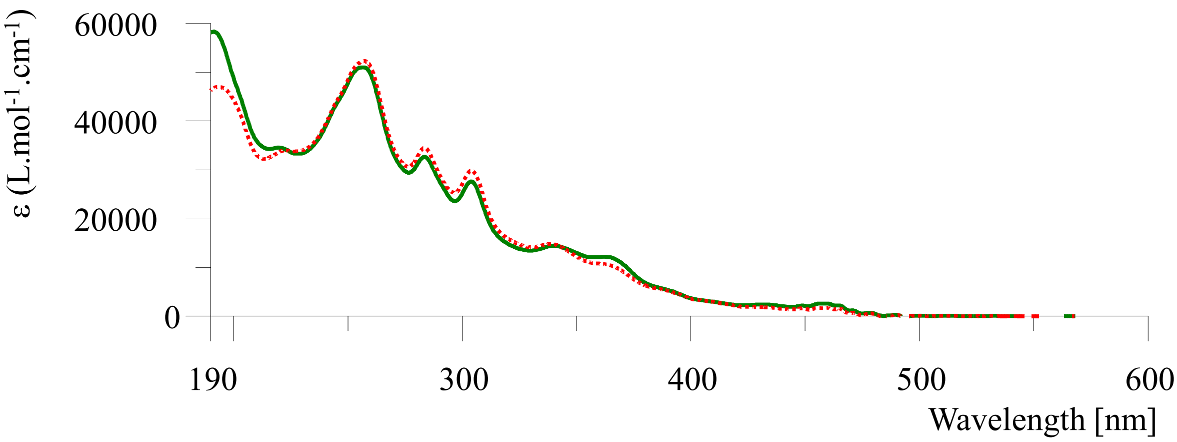

Supplementary Figure 19 | UV-visible spectroscopy. UV-visible spectra for *P* enantiomer (green solid line) and *M* enantiomer (red dotted line) in CH_3CN (0.216 mM, 298 K).

The baseline was always measured for the same solvent and in the same cell as the samples. The spectra are presented without smoothing and further data processing. *P* enantiomer, first eluted on Chiralpak IF: green solid line, concentration = 0.210 mmol·L^{-1} in acetonitrile. *M* enantiomer, second eluted on Chiralpak IF: red dotted line, concentration = 0.216 mmol.L^{-1} in acetonitrile. Acquisition parameters: 0.1 nm as intervals, scanning speed 50 nm/min, band width 2 nm, and 3 accumulations per sample.

NMR spectra

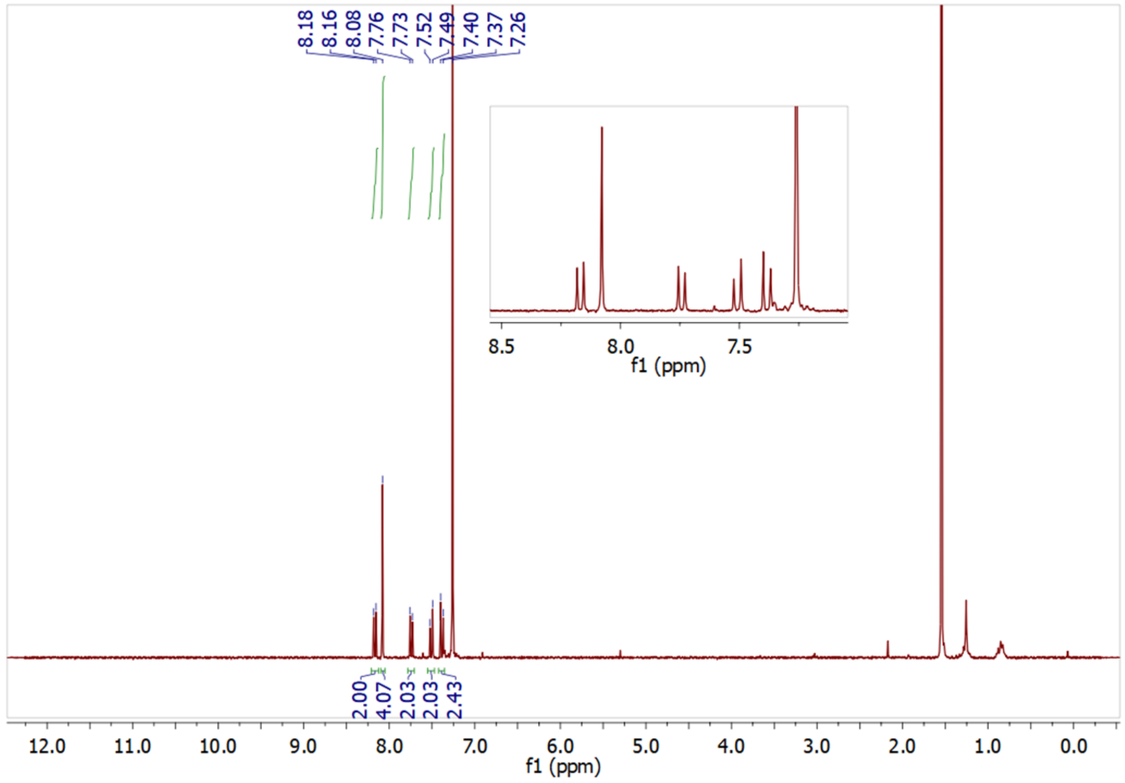

Supplementary Figure 20 | NMR spectroscopy. ^{1}H NMR of (*rac*)-(bis)thiadiazole-[8]helicene.

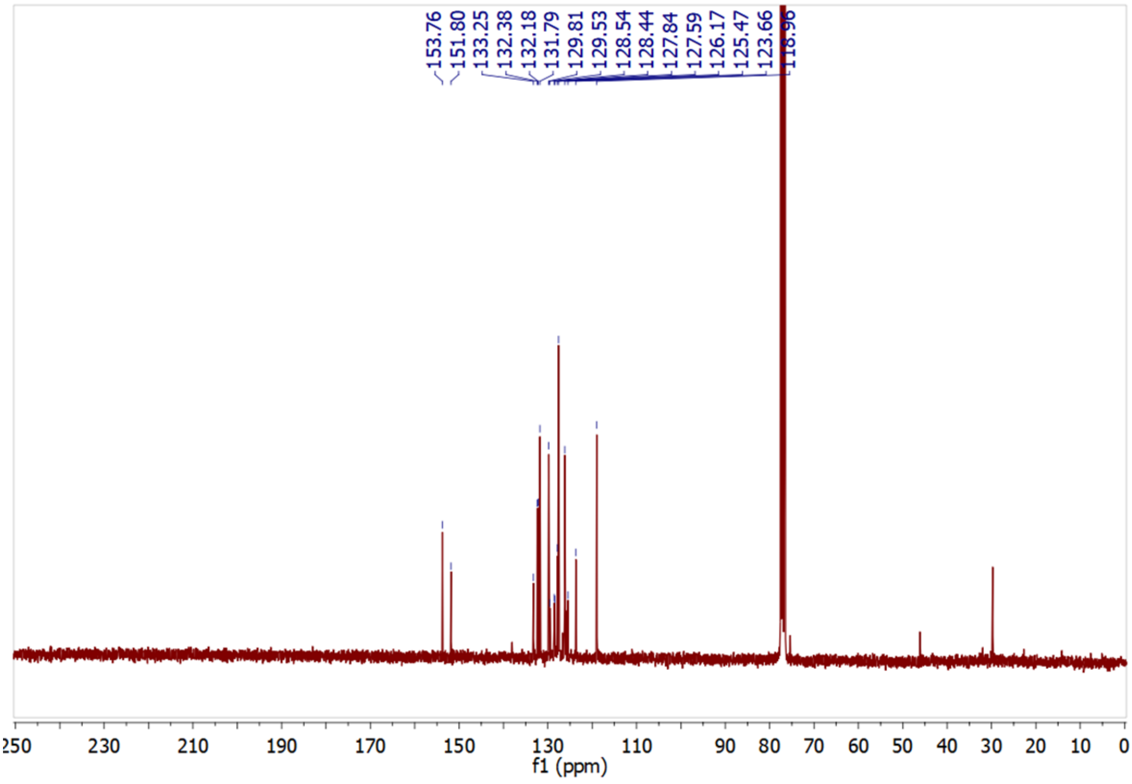

Supplementary Figure 21 | NMR spectroscopy. ^{13}C NMR of (*rac*)-(bis)thiadiazole-[8]helicene

Supplementary References

[1] Görlin, M. et al. Oxygen evolution reaction dynamics, faradaic charge efficiency, and the active metal redox states of Ni–Fe oxide water splitting electrocatalysts. *J. Am. Chem. Soc*. **138**, 5603–5614 (2016).

[2] Farhat, R., Dhainy, J. & Halaoui, L. I. OER Catalysis at Activated and Codeposited NiFe-Oxo/Hydroxide Thin Films Is Due to Postdeposition Surface-Fe and Is Not Sustainable without Fe in Solution. *ACS Catal.* **10**, 20–35 (2020)

[3] Chung, D. Y. et al. Dynamic stability of active sites in hydr(oxy)oxides for the oxygen evolution reaction. *Nature Energy* ***5***, 222–230 (2020).

[4] Mitchell, C., Fayette, M. & Dimitrov, N. Homo- and hetero-epitaxial deposition of Au by surface limited redox replacement of Pb underpotentially deposited layer in one-cell configuration. *Electrochimica Acta* **85**, 450–458 (2012).

[5] Jeyabharathi, C., Zander, M. & Scholz, F. Underpotential deposition of lead on quasi-spherical and faceted gold nanoparticles. *Journal of Electroanalytical Chemistry* **819**, 159–162 (2018).

[6] Mori, K., Murase T. & Fujita, M. One-Step Synthesis of [16]Helicene. *Angew. Chem. Int. Ed.* **54**, 6847–6851 (2015).

[7] Biet, T., Martin, K., Hankache, J., Hellou, N., Hauser, A., Bürgi, T., Vanthuyne, N., Aharon, T., Caricato, M., Crassous, J. & Avarvari, N. Triggering Emission with the Helical Turn in Thiadiazole-Helicenes. *Chem. Eur. J.* **23**, 437–446 (2017).

[8] Sheldrick, G. M. A short history of *SHELX*. *Acta Crystallogr. Sect. A* **64**, 112–122 (2008).

[9] Sheldrick, G. M. *SHELXT* - Integrated space-group and crystal-structure determination. *Acta Crystallogr. Sect. A* **A71**, 3–8 (2015).

[10] Hübschle, C. B., Sheldrick, G. M. & Dittrich, B. *ShelXle*: a Qt graphical user interface for *SHELXL*. *J. Appl. Crystallogr.* **44**, 1281–1284 (2011).

[11] Dolomanov, O. V., Bourhis, L. J., Gildea, R. J., Howard, J. A. K. & Puschmann, H. *OLEX2*: a complete structure solution, refinement and analysis program. *J. Appl. Crystallogr.* **42**, 339–341 (2009).
